# Case report: Post-surgical complication in a case of urethral duplication in a dog

**DOI:** 10.3389/fvets.2022.1013270

**Published:** 2022-11-25

**Authors:** Joana B. Martins, Rita Rosa, Leonor V. Iglésias, Ana Reisinho, Lisa A. Mestrinho

**Affiliations:** ^1^Faculty of Veterinary Medicine, Veterinary Teaching Hospital, University of Lisbon, Lisbon, Portugal; ^2^Faculty of Veterinary Medicine, CIISA – Centre for Interdisciplinary Research in Animal Health, University of Lisbon, Lisbon, Portugal; ^3^Laboratório Associado para Ciência Animal e Veterinária (AL4AnimalS), Lisbon, Portugal

**Keywords:** ectopic urethra, Y-type, perineal approach, revision surgery, dog

## Abstract

Urethral duplication is a rare anomaly observed in veterinary medicine. The surgical techniques described therein are associated with an uneventful recovery. The authors describe a major surgical complication after the correction of urethral duplication in a 2-year-old male Yorkshire terrier. After surgical correction using the perineal approach, the patient developed pollakiuria and urinary retention due to a valve effect caused by the remnant of the dorsal opening of the ectopic urethra. A second procedure, using an abdominopelvic approach, successfully corrected the complication by intraluminal correction of the dorsal urethral wall.

## Case description

### Symptoms at presentation

An intact 2-year-old male Yorkshire, weighting 2.8 kg, presented with a history of aberrant urination and recurrent urinary tract infection. During micturition, the same volume of urine came simultaneously from the anus and urethra.

### Physical exams and lab results

**Figure 1:**
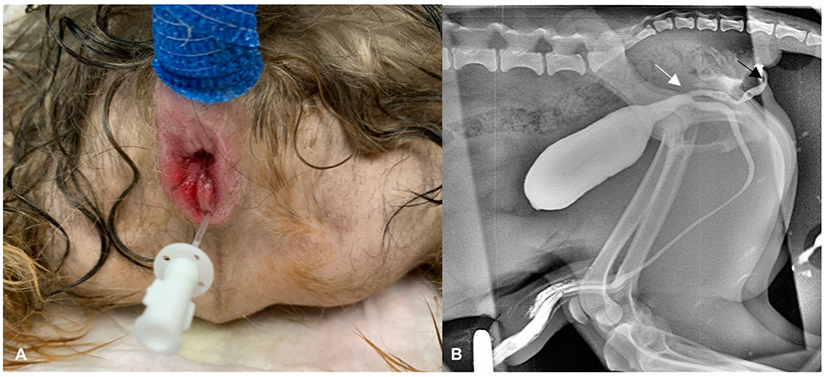
**(A)** Perineal region in a 2-year-old male dog with a urethral duplication. Note the catheterized opening of the ectopic urethra in annus. **(B)** A retrograde cystogram was preformed to identify the location of duplication of the urethra and its length. Note its beginning (white arrow) and ending (black arrow).

**Figure 2:**
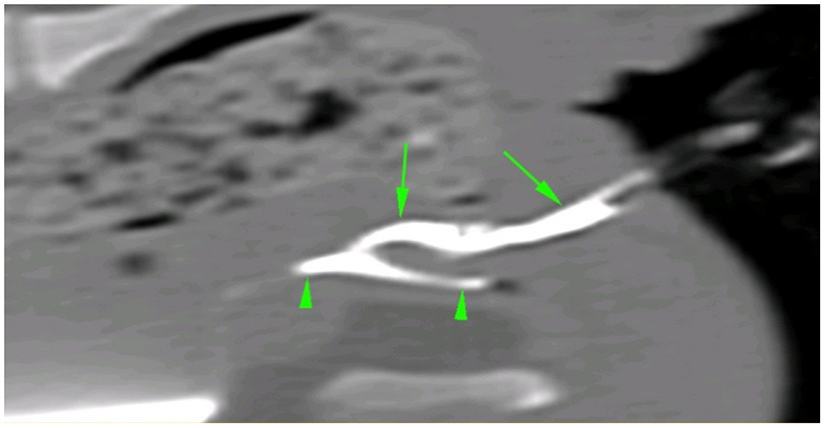
CT sagittal image of the urethral duplication after retrograde injection of iodinated contrast medium in both urethral openings. Note the ectopic urethra (green arrows) and the orthotopic urethra (green arrowhead).

Clinical examination and visualization of the opening at the level of the anus ventrally revealed urine leakage from the anus (Figure 1A). Inguinal testicles were also identified; however, no other abnormalities were noted on physical examination. Hematology and serum biochemistry results were unremarkable except for borderline thrombocytopenia. Computed tomography (CT) revealed a perineal fistula connected to the urethra, compatible with congenital perineal urethral duplication (Figure 2).

Surgery was planned, which included contrast urethrocystography (combination of iodinated contrast—iohexol and saline in a 1:1 proportion, 2 ml/kg BW) prior to the intervention (Figure 1B) and exploration of the ectopic urethral opening with the anus.

### Surgical intervention

Prior to anesthetic induction, the dog was premedicated with methadone (0.3 mg/kg BW, IM) (Semfortan 10 mg/ml, Eurovet Animal Health BV, Netherlands) and acepromazine (0.025 mg/kg BW, IM) (Calmivet 5 mg/ml, Vétoquinol S.A, France), followed by anesthetic induction with midazolam (0.2 mg/kg BW, IV) (Midazolam 15 mg/3ml Labesfal, Portugal) and propofol (2 mg/kg BW, IV) (Propo Vet 10 mg/ml Zoetis, Portugal). The dog was intubated, and the anesthesia was maintained with isoflurane and oxygen. Lactate Ringer's solution was administered at a rate of 5 ml/kg/h. The dog received cefazoline (22 mg/kg BW, IV) (cefazolina, 1000 mg/10 ml Labesfal, Portugal) at induction, and every 90 min during surgery, and robenacoxib (2 mg/kg BW, SC) (Onsior 20 mg/ml Elanco, Portugal). The surgical technique was performed using the perineal approach described by Ralphs and Kramek (1). The dog was placed in sternal recumbency with its hind limbs hanging over the edge of the table and prepared for surgery with both urinary catheters in place (one in each urethral opening) and a purse-string suture in the anus. A 3 cm vertical incision was made over the midline, 0.5 cm below the anus. Blunt and sharp dissection of the external anal sphincter muscle and surrounding tissues was performed along the midline, just ventral to the rectum and dorsal to the pelvic urethra. Dissection was continued until the accessory urethra (with the catheter) was isolated from the surrounding tissues, and communication between the urethra was identified. The accessory urethral length, previously determine with CT and later, intraoperatively, using a sterile measuring tape, was ~2.5 cm (Supplementary Figure 1). At this point, the catheter was withdrawn, and the ectopic structure was double-ligated using a 3-0 monofilament absorbable suture and transected. Closure was performed routinely with a 3-0 monofilament absorbable suture to close the muscular and subcutaneous layers in a simple continuous pattern, and an intradermal suture pattern to close the skin. The recumbency of the dog was changed and the dog was neutered. The dog was discharged that day with instructions to administer amoxicillin-clavulanic acid (Clavubactim; Esteve, Ecuphar, Spain) 12.5 mg/kg body weight (BW), PO, q12 h, for 7 days and paracetamol (Ben-u-ron; Ben Farmacêutica, Farmalabor Produtos Farmacêuticos, S.A., Portugal) 10 mg/kg BW, PO, q12 h, for 5 days for post-surgical analgesia (2).

**Figure 3:**
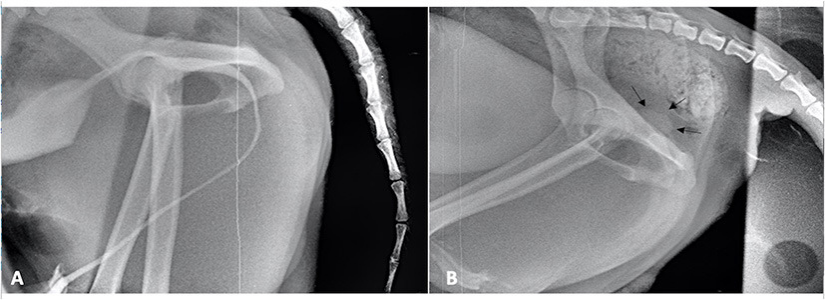
**(A)** Contrast cystogram performed after the first intervention. **(B)** Contrast cystogram performed with manual compression of the bladder. Note the pre-stenotic bulge of the pelvic urethra (black arrows).

**Figure 4:**
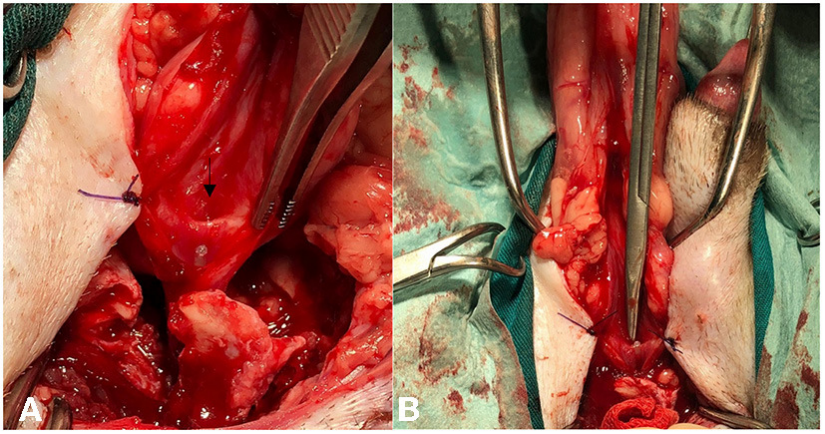
**(A)** Intraoperative image of the opening of the ectopic urethra (black arrow) after urethrotomy. Note the urinary catheter ascending from the pelvic urethra. **(B)** Note the Metzenbaum scissors entering the remnant of the ectopic urethra.

Two days after surgery, the dog presented to the hospital with pollakiuria and urinary retention that started after the surgical intervention, as reported by the owners. There were signs of partial obstruction- a full and tense bladder, pain on palpation, and non-competent urinary leakage. No relief was obtained after administration of buprenorphine. A urinary probe easily passed through the urethra, and this led to immediate relief of obstruction. Retrograde urethrocystography was performed once again, which revealed an apparently normal-sized urethra (initially identified) and no signs of contrast leakage (Figure 3A). The dog was hospitalized for 1 day for pain management and evaluation of micturition. Signs recurred after removal of the urinary probe, and the next day, retrograde cystography with manual compression was performed. During the procedure, a pre-stenotic bulging of the pelvic urethra was identified, which was compatible with dilation of the ectopic urethra remnant leading to a valve effect and dynamic obstruction of the orthotopic urethra (Figure 3B). The dog underwent revision surgery; however, this time, the authors chose abdominopelvic access with an intraluminal approach by ventral urethrotomy. A caudal ventral midline celiotomy was performed. To get adequate exposure of the cranial pelvic urethra, a pubic osteotomy was performed at 3 sites according to Cuddy and McAlinden (3). First, the pre-pubic tendon was incised, and the adductor muscles insertions were elevated from the cranial pubis, exposing the pubic bone. Osteotomies were done with an osteotome and mallet. After retraction of the pubic bone segment to one side, the intrapelvic urethra was mobilized from the pelvic floor. After mobilization of the bladder using stay sutures, a ventral midline incision was made from the caudal bladder to the urethra, at the level of the urethral remnant. The ectopic urethral remnant was identified at the dorsal wall of the orthotopic urethra (Figure 4). The opening of the luminal wall was closed using an acute incision with a 11-sized scalpel blade, followed by a simple interrupted suture using a 6-0 absorbable monofilament (poliglecaprone 25) (Monosyn, BBraun). The orthotopic urethrotomy was closed similarly using a 5/0 similar absorbable monofilament. The bone segment was secured in place with orthopedic wire, the adductor muscles were repositioned and sutured, as well as the pre-pubic ligament using polydioxanone (Monoplus, BBraun). The remaining tissues were closed routinely.

### Outcome and follow-up

Postoperative recovery was uneventful, without recurrence of clinical signs. Six months after the latest surgical intervention, the patient presented with pollakiuria and urinary incontinence. Urine culture with antibiotic sensitivity testing was performed, and Enterococcus faecium was identified, which was sensitive only to sulfamethoxazole and trimethoprim. Antibiotic therapy was initiated with this combination (Bactrim, Roche Pharma, Grenzach-Wyhlen, Germany) 15 mg/kg BW, PO, q12 h for 15 days, and phenylpropanolamine (Propalin, Vetoquinol, France) 1.2 mg/kg BW, PO, q12 h. The dog continued to show signs of occasional uncontrolled urinary micturition associated with stress. Therefore, the frequency of phenylpropanolamine administration was corrected to q8 h and maintained for 2 months. Urethropexy was proposed; however, it was refused by the owner. Eight months later, the owner reported an improvement in the clinical signs of the dog. Uncontrolled urinary micturition related to stress occurred, although less frequently.

## Discussion

Relevant complications associated with the surgical correction of urethral duplication must be reported as they can contribute to a better management of future cases.

This rare congenital condition has likely multifactorial etiology, mostly found in males (3, 4). Different anatomical variations can occur; however, in most cases, there is mainly an ectopic urethra that can emerge from the neck of the bladder or from the primary urethra. It can communicate again with the urethra, end in a blind pouch, or communicate with the rectum, anus, or skin (5). The anatomical diversity and lack of a uniform embryologic theory have led to multiple classifications of this abnormality. In this case report, the dog presented a Y-type duplication (type IIA2), complete, with external communication (6, 7). The classification proposed by Effmann et al. (7) acknowledges three types of duplications for humans, latter adapted in one previous case report (8). Type I includes blind, incomplete urethral duplications, and type II include complete urethral duplications. In type IIA1, two non-communicating urethras emerge independently from the bladder. In type IIA2, the second urethra arises from the first and courses independently into a second meatus, the IIB type includes cases in which the two urethras unite distally into one common channel. Type III represents partial or complete caudal duplication.

Urethral duplication can either be asymptomatic or present with several signs, including inflammation, incontinence, urinary obstruction, recurrent urinary infection, and double urinary stream (5, 9). Most of these signs were observed in this case except for incontinence, which was only observed after urinary tract infection. Cryptorchidism was also identified, and possibly associated (9, 10). Other commonly associated anomalies include penile, vaginal, uterus, and colon duplications, vesicoureteral reflux, renal agenesis and ectopia, renal malrotation, multicystic-dysplastic kidneys, duplex kidney and ureter, vertebral anomalies, anorectal formations, tracheoesophageal anomalies, and bifid scrotum (9–12).

Although surgery of type IIA2 urethral duplication has been previously reported in dogs (1, 6, 8, 13, 14) no major post operative complications have been described. The authors report, for the first time, a major postoperative complication after urethral duplication correction in a dog. Complications are frequent with similar procedures in children. Specifically, type IIA2 surgical correction is associated with a complication rate as high as 75%, with the patients being implicated in a series of surgical procedures (average of more than four procedures per patient), especially when the ectopic urethra is the most functional (9, 15). Y-type duplications can be also classified into pure, steno-atretic, and abortive forms, where the pure forms present a functional orthotopic and ectopic urethra, which was also observed in this case report (7, 10). From what is known in the human medicine literature, in most cases, the functional urethra is the ectopic one, whereas the orthotopic is poorly developed (10, 15). In the current case, both urethras appeared equally developed with similar calibers. Although, the diameter can be an important factor to consider, micturition volume of both urethral openings might also contribute to the assessment of the ectopic urethra. Unfortunately, this was not evaluated in our case. Anatomical differences between the urethras in dogs have not been previously explored, however, revisiting two reports, the ectopic urethra appeared to be underdeveloped and of inferior caliber when compared with the orthotopic one (8, 13).

The surgical options reported in human surgery literature include reconstruction or excision. Reconstruction includes the use of mucosal grafts or local tissue with mobilization of the ectopic urethra by perineal and/or transpubic approaches (5, 15, 16). Excision of the ectopic urethra is chosen when it is considered less functional, and the orthotopic urethra is normal in diameter and function (15–18). The authors believe that this major complication was a consequence of leaving a remnant of a functional ectopic urethra. In this case, both ectopic and orthotopic urethras were equally functional. The mechanism proposed by the authors is that the remnant of the ectopic urethra, when filled with urine, bulges and acts like a valve compressing the normal urethra and blocks normal urine flow. This theory is supported by the second contrast cystogram performed with manual compression, in which pre-stenotic bulging of the urethra can be seen. This mechanism has been reported in human medical literature to explain the outflow obstruction observed in children with this type of malformation (5). Looking at previous case reports in dogs, a 1 cm remnant of ectopic urethra was not related to any complications (8). This lack of complication might be related with the small diameter of the ectopic urethra compared with the orthotopic one, which was the opposite in our case.

The theory proposed by the authors of a valve effect causing outflow obstruction is also supported by other observations. First, there were no resistance while placing the urethral catheter. In this case we decided not to leave a urinary catheter in the post operatory period for two reasons: no expected obstruction/reduction of the luminal diameter and its presence could promote inflammation (3). Second, post operative pain was assured by paracetamol (2), and confirmed clinically in the immediate post operative period before discharge. Additionally, when the animal returned with urinary retention and pollakiuria, pain was also assessed and treated with buprenorphine. Pain treatment did not provide any relief.

The stress induced incontinence observed at the long term could be related with several factors. First, the urinary infection. This event could be a result of an undiagnosed urinary colonization, which evolved to infection slowly, or could be a new event. Treatment of the infection contributed to attenuation of the stress induced incontinence. Second, an incompetent orthotopic urethra or a possible hormone responsive incontinence are other causes to consider. The latest cause usually leads to micturition during sleep or rest (19), the opposite to what was observed in this case. Finally, iatrogenic nerve damage (pelvic, pudendal, or hypogastric) (20). This event would most likely occur in the first surgical procedure, since in the abdominopelvic approach, the urethra was acceded ventrally, and briefly dissected, making nerve damage less likely to occur. Regardless, if iatrogenic nerve damage occurred, the dog would not be completely recovered and with no incontinence for over a period of 6 months. Urodynamic studies would be necessary to further study this case but were not pursued by the owner.

This case report suggests that it is crucial to evaluate the type of duplication and functions of both urethras to choose the most appropriate surgical technique and avoid complications. In this case, the perineal approach described previously (1) was not sufficient to correct the malformation, since there was the need to completely remove the ectopic urethra. The abdominal approach should be considered as part of a combined approach in this and possible future cases after accurately access anatomy and function of both urethras.

## Data availability statement

The raw data supporting the conclusions of this article will be made available by the authors, without undue reservation.

## Ethics statement

Ethical review and approval was not required for the study of animals in accordance with the local legislation and institutional requirements. Written informed consent was obtained from the owner of the animal involved in this case report.

## Author contributions

JM collected all clinical data and wrote the initial draft. RR, LI, AR, and LM performed the diagnostics and all medical and surgical procedures. All authors contributed to the writing of the manuscript, after discussion of the results, revised the manuscript, contributed equally to the final version, and agreed to the copyright conditions.

## Funding

This work was funded by FCT—Fundação para a Ciência e Tecnologia, grant UIDB/00276 /2020, from CIISA—Centro de Investigação Interdisciplinar de Sanidade Animal, Faculdade de Medicina Veterinária, Universidade de Lisboa, and LA/P/0059/2020 - AL4AnimalS, Portugal.

## Conflict of interest

The authors declare that the research was conducted in the absence of any commercial or financial relationships that could be construed as a potential conflict of interest.

## Publisher's note

All claims expressed in this article are solely those of the authors and do not necessarily represent those of their affiliated organizations, or those of the publisher, the editors and the reviewers. Any product that may be evaluated in this article, or claim that may be made by its manufacturer, is not guaranteed or endorsed by the publisher.

## Supplementary material

Supplementary Figure 1 Intraoperative image after dissection around the catheterized ectopic urethra (with ~2.5 cm of length) from the first surgery.
