# Supplementary figures and images for: Case report: Post-surgical complication in a case of urethral duplication in a dog
Source: Front Vet Sci. 2022 Nov 25;9:1013270. doi: 10.3389/fvets.2022.1013270 (PMC9732570; doi:10.3389/fvets.2022.1013270)

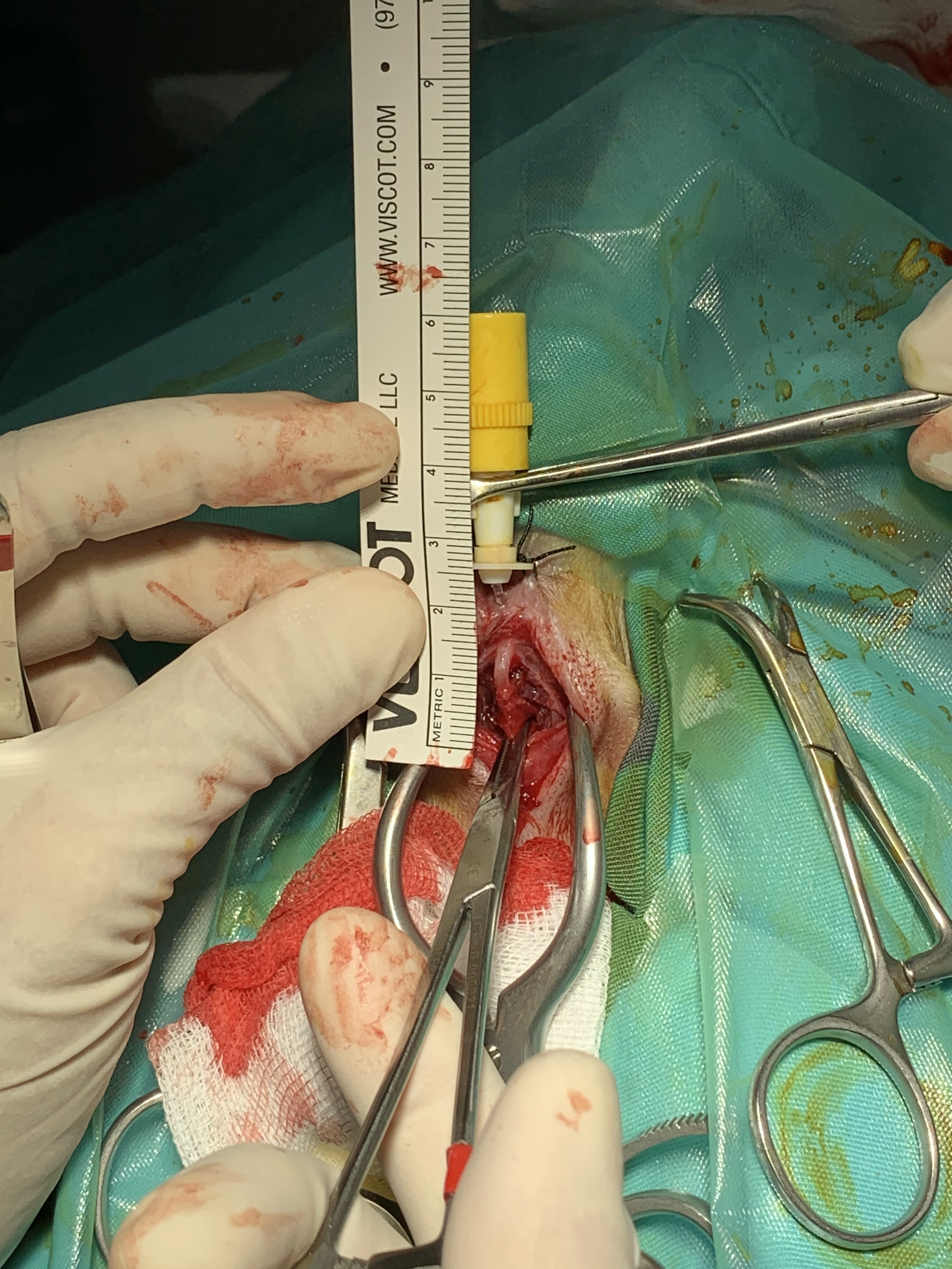

Supplement: Supplementary Figure 1 — Intraoperative image after dissection around the catheterized ectopic urethra (with ~2.5 cm of length) from the first surgery. [file Image_1.JPEG]
